# Cost-Effectiveness Analysis of Diagnostic Options for *Pneumocystis* Pneumonia (PCP)

**DOI:** 10.1371/journal.pone.0023158

**Published:** 2011-08-15

**Authors:** Julie R. Harris, Barbara J. Marston, Nalinee Sangrujee, Desiree DuPlessis, Benjamin Park

**Affiliations:** 1 Mycotic Diseases Branch, National Center for Emerging and Zoonotic Infectious Diseases, Centers for Disease Control and Prevention, Atlanta, Georgia, United States of America; 2 Global AIDS Program, Center for Global Health, Centers for Disease Control and Prevention, Atlanta, Georgia, United States of America; 3 Parasitology Reference Unit, National Institute for Communicable Diseases, National Health Laboratory Service, Johannesburg, South Africa; New York State Health Department and University at Albany, United States of America

## Abstract

**Background:**

Diagnosis of *Pneumocystis jirovecii* pneumonia (PCP) is challenging, particularly in developing countries. Highly sensitive diagnostic methods are costly, while less expensive methods often lack sensitivity or specificity. Cost-effectiveness comparisons of the various diagnostic options have not been presented.

**Methods and Findings:**

We compared cost-effectiveness, as measured by cost per life-years gained and proportion of patients successfully diagnosed and treated, of 33 PCP diagnostic options, involving combinations of specimen collection methods [oral washes, induced and expectorated sputum, and bronchoalveolar lavage (BAL)] and laboratory diagnostic procedures [various staining procedures or polymerase chain reactions (PCR)], or clinical diagnosis with chest x-ray alone. Our analyses were conducted from the perspective of the government payer among ambulatory, HIV-infected patients with symptoms of pneumonia presenting to HIV clinics and hospitals in South Africa. Costing data were obtained from the National Institutes of Communicable Diseases in South Africa. At 50% disease prevalence, diagnostic procedures involving expectorated sputum with any PCR method, or induced sputum with nested or real-time PCR, were all highly cost-effective, successfully treating 77–90% of patients at $26–51 per life-year gained. Procedures using BAL specimens were significantly more expensive without added benefit, successfully treating 68–90% of patients at costs of $189–232 per life-year gained. A relatively cost-effective diagnostic procedure that did not require PCR was Toluidine Blue O staining of induced sputum ($25 per life-year gained, successfully treating 68% of patients). Diagnosis using chest x-rays alone resulted in successful treatment of 77% of patients, though cost-effectiveness was reduced ($109 per life-year gained) compared with several molecular diagnostic options.

**Conclusions:**

For diagnosis of PCP, use of PCR technologies, when combined with less-invasive patient specimens such as expectorated or induced sputum, represent more cost-effective options than any diagnostic procedure using BAL, or chest x-ray alone.

## Introduction

*Pneumocystis jirovecii* causes a fungal pneumonia (PCP) affecting HIV-infected and other immunocompromised persons worldwide [1]. Although highly active anti-retroviral therapy (HAART) and PCP prophylaxis, usually with cotrimoxazole (CTX), have reduced the burden of PCP among AIDS patients in developed countries [2], [3], [4], [5], PCP remains an important cause of HIV-related morbidity and mortality throughout much of the developing world [1]. The prevalence of PCP among HIV-infected African children with pneumonia ranges from 10 to 49% [6], [7], [8], [9], [10], with mortality as high as 80% [11]. Among African adults, in whom the disease is often misdiagnosed as smear-negative TB [12], [13], [14], increases in PCP diagnoses have been noted during the past 15 years [15], [16], [17], [18], [19], [20]. In Southeast Asia, PCP prevalence among HIV-infected children and adults with pneumonia has been reported to be as high as 66% [21], [22], [23]. Among HIV-uninfected persons, those at risk of PCP include persons receiving immunosuppressive therapies, such as renal transplant patients (estimated cumulative PCP incidence: 0.4%) [24], [25], patients undergoing immunosuppressive therapy for connective tissue disorders [26], and children with chronic lung diseases [27]. Mortality from PCP among HIV-uninfected patients can be as high as 40% [1].

Laboratory-based diagnosis of PCP is a two-step procedure, involving specimen collection and pathogen detection (referred to hereafter as the ‘diagnostic procedure’). Specimens can be collected from oral washes (OW), expectorated (ES) or induced (IS) sputum, tracheal secretions, broncho-alveolar lavage (BAL), or transbronchial biopsies from patients; the latter two require bronchoscopy. Several different methods can be employed for pathogen detection on all specimen types, including immunofluorescence microscopy (IFA), *Pneumocystis* cyst wall stains [Toluidine Blue O (TBO) and calcofluor white (CW)], *Pneumocystis* trophozoite stains [Grocott's methenamine silver stain (GMS), Diff-Quick (DQ), and Papanicolaou], or single-round polymerase chain reaction (PCR), nested PCR (nPCR), or quantitative real-time PCR (rtPCR) [15], [28], [29], [30] to amplify genomic DNA.

However, accurate diagnosis of PCP poses multiple challenges. While the procedures to obtain oral washes and sputa are less invasive than that for BAL, they are also less effective at obtaining sufficient numbers of organisms for visualization with diagnostic stains. In contrast, the cost and invasiveness of bronchoscopy and the technical skill it requires render it unfeasible in many areas of the world. Pathological interpretations of stained slides are subjective and nonspecific; sensitivity is dependent on the burden of pathogen in the sample, the specimen type employed, and the skill and experience of the technician examining the sample. Among the pathogen detection methods, the most sensitive is PCR; however, it may be technologically and economically impractical for much of the developing world. Because of these factors, clinicians often use chest x-rays and clinical evaluations as the sole diagnostic method for *Pneumocystis* pneumonia. Although many studies have evaluated the test characteristics of different diagnostic methods [28], [29], [30], [31], [32], [33], [34], [35], [36], [37], [38], [39], [40], [41], [42], [43], [44], [45], [46], [47], [48], [49], [50], [51], [52], [53], [54], [55], [56], [57], [58], [59], [60], [61], [62], [63], [64], [65], [66], [67], [68], [69], [70], [71], [72], [73], [74], [75], [76], [77], [78], [79], [80], comparisons of costs and outcomes have not been presented.

This report reviews available diagnostic procedure options for PCP, as well as the cost-effectiveness of each option as a function of procedural cost, sensitivity, and specificity. The outcome measures of interest are the proportion of PCP patients successfully treated and the cost per life-year gained. The analysis is considered from the perspective of the health care payer in developing countries (typically the government). The results should help guide decision-making with respect to diagnostic options for PCP in the developing world.

## Methods

### Assumptions

#### Patient population and setting

This analysis is conducted among ambulatory HIV-infected patients in South Africa.

#### Test qualities and diagnostic costs

Estimates of sensitivity and specificity of diagnostic procedures used in the model are shown in Table 1 and are based on reports from the literature (chest x-ray alone, oral wash with DQ, PCR, nested PCR, and rtPCR; expectorated sputum with GMS, TBO, CW; induced sputum with DQ, GMS, TBO, IS, IFA, PCR, nPCR; BAL with DQ, GMS, TBO, CW, IFA, PCR, nPCR, rtPCR) [29], [30], [31], [36], [38], [39], [40], [44], [47], [48], [49], [50], [52], [54], [55], [57], [59], [60], [61], [62], [63], [64], [65], [66], [67], [68], [69], [70], [71], [72], [73], [74], [75], [76], [77], [78], [79], [80], [81], [82], [83], [84], or estimated by the authors (oral wash with IFA, GMS, CW, TBO; expectorated sputum with DQ, IFA, PCR, nPCR, or rtPCR; induced sputum with rtPCR). Although all of the above-referenced literature was consulted, for diagnostic procedures involving any form of PCR, only studies which targeted the mitochondrial large subunit ribosomal RNA were used for estimations of sensitivity and specificity [28], [29], [36], [48], [51], [54], [55], [57], [63], [64], [67], [68], [69], [71], [72], [75], [76], [78], [85], [86]. For tests for which data did not exist in the literature, estimations of test characteristics were based on interpolation and pre-existing knowledge of the sensitivity and specificity of other tests in the same diagnostic category (e.g., we assumed that expectorated sputum with DQ, for which we did not find published reports, would be intermediate in sensitivity between oral wash with DQ and induced sputum with DQ, for which we were able to reference published reports.)

**Table 1:** Model inputs and costs: Sensitivity and specificity of diagnostic procedures, based on estimates derived from existing studies (see text) or, when reference studies not available, from author estimation.

Diagnostic	Specimen collection	Sensitivity	Specificity	Cost (USD)
CXR	None	0.86	0.40	$40.00
DQ	Oral wash	0.30	1.00	$2.32
	Expectorated sputum	0.60	1.00	$2.22
	Induced sputum	0.75	1.00	$8.72
	Bronchoalveolar lavage	0.75	1.00	$77.12
GMS	Oral wash	0.30	1.00	$4.21
	Expectorated sputum	0.52	0.95	$4.11
	Induced sputum	0.70	0.96	$10.61
	Bronchoalveolar lavage	0.82	0.98	$79.01
TBO	Oral wash	0.30	1.00	$0.93
	Expectorated sputum	0.71	1.00	$0.83
	Induced sputum	0.75	1.00	$7.33
	Bronchoalveolar lavage	0.80	1.00	$75.73
CW	Oral wash	0.30	1.00	$2.94
	Expectorated sputum	0.33	1.00	$2.84
	Induced sputum	0.57	1.00	$9.34
	Bronchoalveolar lavage	0.78	1.00	$77.74
IFA	Oral wash	0.30	1.00	$20.79
	Expectorated sputum	0.50	1.00	$20.69
	Induced sputum	0.81	1.00	$27.19
	Bronchoalveolar lavage	1.00	1.00	$95.59
PCR	Oral wash	0.71	0.99	$8.78
	Expectorated sputum	0.85	0.99	$8.68
	Induced sputum	0.94	0.99	$15.18
	Bronchoalveolar lavage	1.00	0.94	$83.58
nPCR	Oral wash	0.83	1.00	$10.32
	Expectorated sputum	0.91	1.00	$10.22
	Induced sputum	1.00	1.00	$16.72
	Bronchoalveolar lavage	1.00	0.89	$85.12
rtPCR	Oral wash	0.89	0.94	$13.84
	Expectorated sputum	0.92	0.94	$13.74
	Induced sputum	0.95	0.90	$20.24
	Bronchoalveolar lavage	0.99	0.80	$88.64

CXR: Chest x-ray; DQ: Diff-Quick; GMS: Grocott's Methenamine Silver Stain; TBO: Toluidine Blue O; CW: Calcofluor white stain; IFA: Immunofluorescence; PCR: Polymerase chain reaction; nPCR: nested PCR; rtPCR: real-time (quantitative) PCR.

Estimates of costs include required materials and personnel time (Tables S1 and S2). Total costs for diagnostic procedures are included in Table 1. Estimated salaries for laboratory and health care workers are available in Appendix S1. Except where stated, all cost and time estimates were provided by the National Institute of Communicable Diseases in South Africa.

#### Others

The value referred to as ‘prevalence’ refers specifically to the prevalence of disease among patients with signs and symptoms of PCP who would normally warrant testing at a given hospital or clinic. It does not refer to the population prevalence of disease. This value will differ regionally; some hospitals or clinics might test all patients with respiratory disease and negative AFB smears, while others will test only patients who have a chest x-ray typical for PCP.

Three models are also presented, with prevalences set at 5%, 20%, and 50%. Treatment failure, whether related to insufficient adherence to treatment or breakthrough infections during treatment to which the patient is adherent, is assumed to occur among 10% of patients (Table S3).

Treatment costs are based on a single, 21-day regimen with oral CTX (Table S3). Patients are assumed to not be taking CTX at the time of diagnosis.

#### Life-years gained

In studies carried out before the year 2000, median survival time after AIDS diagnosis among patients in developing countries not on antiretroviral therapy was calculated to be approximately one year [87]. In the absence of treatment, PCP is generally accepted to lead to rapid death. Therefore, we assumed that diagnosis and appropriate treatment led to a single life-year gained among patients with PCP, compared with patients who were not diagnosed correctly.

#### Model flow

**Figure 1:**
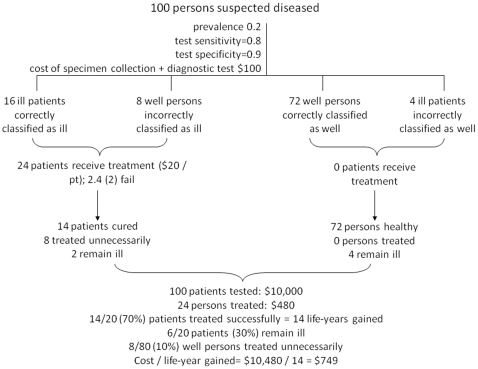
Model flow. ‘Ill patients’ refers to patients with PCP. ‘Well persons’ refers to persons without PCP, regardless of their health status otherwise. Patients successfully treated are assumed to gain one life-year.

An example of model flow with sample values is depicted in Figure 1. ‘Ill patients’ refers to patients with PCP; ‘well persons’ refers to persons without PCP (although persons in this group likely have another illness, since they are undergoing testing). At a given PCP prevalence among persons tested, the number of ill patients correctly diagnosed is calculated as the sensitivity of the diagnostic procedure (Table 1) multiplied by the total number of ill patients. The number of well persons incorrectly classified as ill is equal to the total number of well persons, minus the procedural specificity (Table 1) multiplied by the total number of well persons. The total number of persons classified as ill is the sum of these values. Total diagnostic procedural costs are calculated as a sum of the health care worker and laboratory staff costs and material costs for the specimen collection and the diagnostic test procedures (Table 1 and Tables S1 and S2).

All persons diagnosed as PCP-positive (correctly or incorrectly) are assumed to receive a full course of treatment. Treatment failure rates are considered as a combination of failure-to-adhere and breakthrough infection rates (Table S3). The number of patients who fail treatment is equal to the number of ill patients correctly classified as ill who undergo treatment, multiplied by the treatment failure rate. Because each patient is assumed to gain a single year of life from correct treatment, total life-years gained is equal to the number of ill patients correctly diagnosed minus those for whom treatment did not successfully treat infection (Figure 1).

The proportion of ill patients successfully treated is represented by the number of patients successfully treated divided by the number ill, while the proportion unnecessarily treated is equal to the number of well persons treated divided by the total number of well persons. Total treatment costs are equal to the total number of well persons and ill patients who receive treatment, multiplied by the estimated treatment cost. Finally, the total diagnostic and treatment cost per life-year gained (the cost-effectiveness ratio) is equal to the sum of the total diagnostic costs and the total treatment costs, divided by the number of ill patients successfully treated. The incremental cost-effectiveness ratios of the most effective options were then calculated.

Relapse rates are not considered. Start-up and indirect costs (building costs, laboratory equipment purchase, electricity, training) are also not considered, as they will differ greatly by region and available pre-existing infrastructure.

#### Sensitivity analyses

Sensitivity analyses were performed by varying specific parameters, including treatment costs, treatment failure rates, and costs of diagnostic procedures, over a range of plausible values to determine the impact of uncertainty in the data, and the robustness of results.

## Results

Results from the analyses are presented in Table 2. Nearly all laboratory-based diagnostic procedures have an estimated specificity >90%; thus, few false positives occur even when employing the least sensitive diagnostic procedures (Table 1) and specificity does not contribute to the cost-effectiveness of most diagnostic procedures (the exception is chest x-ray). In general, diagnostic procedures that resulted in the highest proportion of patients successfully treated involved PCR, nPCR, or rtPCR, regardless of the specimen type used for diagnosis. Only one non-PCR-based laboratory diagnostic procedure (IFA with BAL) resulted in successful treatment of ≥75% of patients, while all PCR-based diagnostic procedures except one (PCR with OW) resulted in successful treatment of ≥75% of patients (Table 2).

**Table 2:** Model outputs: Average Cost effectiveness ratio (ACER) (total diagnostic and treatment cost per life-year gained) for each diagnostic procedure.

Diagnostic	Specimen	% of patients successfully treated	ACER (5% prevalence)	ACER (20% prevalence)	ACER (50% prevalence)
CXR	None	77.0%	$1,077	$270	$109
DQ	Oral wash	27.0%	$175	$46	$20
	Expectorated sputum	54.0%	$85	$24	$11
	Induced sputum	67.5%	$261	$68	$29
	Bronchoalveolar lavage	67.5%	$2,288	$574	$232
GMS	Oral wash	27.0%	$315	$81	$34
	Expectorated sputum	46.8%	$184	$48	$21
	Induced sputum	63.0%	$343	$88	$37
	Bronchoalveolar lavage	73.8%	$2,146	$539	$217
TBO	Oral wash	27.0%	$72	$20	$10
	Expectorated sputum	64.3%	$29	$10	$6
	Induced sputum	67.5%	$220	$57	$25
	Bronchoalveolar lavage	72.0%	$2,107	$529	$213
CW	Oral wash	27.0%	$221	$58	$25
	Expectorated sputum	29.7%	$194	$51	$22
	Induced sputum	51.3%	$367	$94	$39
	Bronchoalveolar lavage	70.2%	$2,218	$557	$225
IFA	Oral wash	27.0%	$1,543	$388	$157
	Expectorated sputum	45.0%	$923	$233	$95
	Induced sputum	72.9%	$749	$190	$78
	Bronchoalveolar lavage	90.0%	$2,127	$534	$216
PCR	Oral wash	63.9%	$279	$72	$31
	Expectorated sputum	76.5%	$231	$60	$26
	Induced sputum	84.6%	$363	$93	$39
	Bronchoalveolar lavage	90.0%	$1,864	$468	$189
nPCR	Oral wash	74.7%	$279	$72	$31
	Expectorated sputum	81.9%	$253	$65	$28
	Induced sputum	90.0%	$375	$96	$40
	Bronchoalveolar lavage	90.0%	$1,901	$477	$193
rtPCR	Oral wash	80.1%	$353	$90	$38
	Expectorated sputum	82.8%	$339	$87	$36
	Induced sputum	85.5%	$483	$123	$51
	Bronchoalveolar lavage	89.1%	$2,005	$503	$203

CXR: Chest x-ray; DQ: Diff-Quick; GMS: Grocott's Methenamine Silver Stain; TBO: Toluidine Blue O; CW: Calcofluor white stain; IFA: Immunofluorescence; PCR: Polymerase chain reaction; nPCR: nested PCR; rtPCR: real-time (quantitative) PCR.

At a disease prevalence of 50%, eight diagnostic procedures had average cost-effectiveness ratios ≤$25 per life-year gained; among these, the most effective (in terms of proportion of PCP patients successfully treated) were IS/TBO, ES/TBO, and ES/DQ (successfully treating 68%, 64%, and 54% of PCP patients, respectively, at $25, $6, and $11 per life-year gained, respectively). Fifteen procedures had an average cost-effectiveness ratio of $26–$100 per life-year gained; among these, the most effective procedures were IS/nPCR, IS/rtPCR, and IS/PCR (resulting in successful treatment of 90%, 86%, and 85% of PCP patients, respectively, at $40, $51, and $39 per life-year gained). Above $100 per life-year gained, the most effective procedures were BAL/nPCR, BAL/IFA, and BAL/PCR, all successfully treating 90% of patients and costing $193, $216, and $189 per life-year gained, respectively. Using a chest x-ray alone for diagnosis resulted in an average cost-effectiveness ratio of $109 per life-year gained and the successful treatment of 77% of patients. Although total costs varied with disease prevalence, relative costs and cost-effectiveness ratios did not (Table 2).

**Figure 2:**
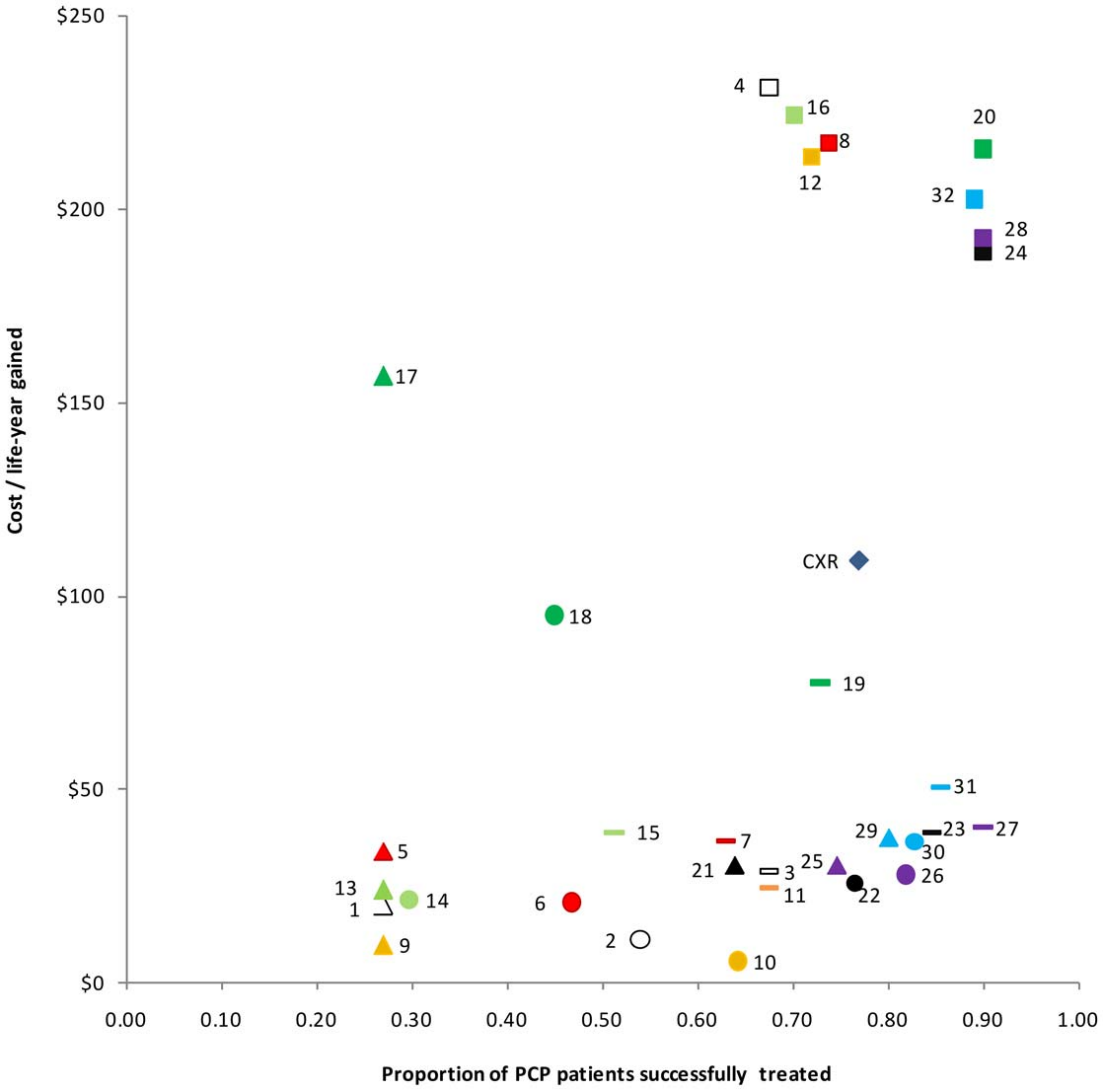
Scatterplot showing total costs for diagnostic procedures and treatment, per life-year gained, at 50% prevalence among population tested. Triangles represent procedures involving oral washes; circles represent procedures involving expectorated sputum; lines represent procedures involving induced sputum; and squares represent procedures involving BAL. All white data points outlined in black indicate procedures using the Diff-Quick test (1–4); red indicates procedures using GMS (5–8); orange indicates procedures using TBO (9–12); light green indicates procedures using calcofluor white (13–16); dark green indicates procedures using IFA (17–20); black indicates procedures using PCR (21–24); purple indicates procedures using nPCR (25–28); and blue indicates procedures using rtPCR (29–32). Figure 2 Legend: 1: DQ/OW; 2: DQ/ES; 3: DQ/IS; 4: DQ/BAL; 5: GMS/OW; 6: GMS/ES; 7: GMS/IS; 8: GMS/BAL; 9: TBO/OW; 10: TBO/ES; 11: TBO/IS; 12: TBO/BAL; 13: CW/OW; 14: CW/ES; 15: CW/IS; 16: CW/BAL; 17: IFA/OW; 18: IFA/ES; 19: IFA/IS; 20: IFA/BAL; 21: PCR/OW; 22: PCR/ES; 23: PCR/IS; 24: PCR/BAL; 25: nPCR/OW; 26: nPCR/ES; 27: nPCR/IS; 28: nPCR/BAL; 29: rtPCR/OW; 30: rtPCR/ES; 31: rtPCR/IS; 32: rtPCR/BAL; CXR: chest x-ray.

The scatterplot in Figure 2 demonstrates the relationship between cost and outcomes of the individual diagnostic procedures. The best outcomes (the highest proportion of patients successfully treated as a result of proper diagnosis and subsequent treatment) are achieved using procedures on the right-hand side of the plot, while the least expensive procedures (per life-year gained) are on the bottom of the plot. The most cost-effective procedures, then, are those that cluster in the bottom right. These procedures include any combination of induced sputum with PCR, nPCR, or rtPCR; expectorated sputum with nPCR or rtPCR; and oral wash with rtPCR, all resulting in the successful treatment of 80–90% of PCP patients at relatively reduced costs per life-year gained (Figure 2). The most expensive procedures per life-year gained, represented as squares in the top right of the figure, all involve BAL, and result in 68–90% of patients being successfully treated. It is worth noting that the diagnostic procedure with the highest cost per life-year gained (DQ/BAL, at $232 per life-year gained) results in similar proportions of patients successfully treated (68%) to the diagnostic procedure with the lowest cost per life-year gained (ES/TBO, successfully treating 64% of PCP patients at $6 per life-year gained).

### Incremental cost-effectiveness of selected options

We evaluated incremental cost-effectiveness among diagnostic options which resulted in successful treatment of at least 2/3 (67%) of PCP patients. After excluding both strongly dominated (less effective and more expensive) and weakly dominated (equally effective but more expensive, or equal in cost but less effective) options, five procedures remained for inclusion in an incremental cost-effectiveness analysis: induced sputum with TBO, PCR, or nPCR; and expectorated sputum with nPCR or rtPCR (Table 3). Using nPCR with expectorated or induced sputum provided a relatively higher benefit at lower cost ($43 and $60 for each additional life-year gained, respectively), compared with the next-least-effective procedure (Table 3).

**Table 3:** Incremental cost-effectiveness of a subset of diagnostic procedures for *Pneumocystis* pneumonia, assuming 50% disease prevalence.

Specimen collection and diagnostic procedure	% of patients successfully treated	Average cost per life-year gained	Cost per additional life-year gained, compared with next-least-effective procedure
IS/TBO	0.68	$25	$N/A
ES/nPCR	0.82	$28	$43
ES/rtPCR	0.83	$36	$804
IS/PCR	0.85	$39	$156
IS/nPCR	0.90	$40	$60

IS: Induced sputum; ES: expectorated sputum; TBO: Toluidine Blue O; PCR: polymerase chain reaction; nPCR: nested PCR; rtPCR: real-time (quantitative) PCR.

### Sensitivity analysis

Variations in the cost of the diagnostic procedure had the most impact on cost per life-year gained in sensitivity analyses. Reducing the cost of the diagnostic procedures by 50% led to an approximate 50% reduction in cost per life-year gained, while doubling it led to approximately a two-fold increase in cost per life-year gained (Table S4). Modifying other factors, including the procedural sensitivity, specificity, treatment failure rates, or treatment cost had little effect on the overall cost per life-year gained. None of the analyses examined affected the relative cost-effectiveness of the diagnostic procedures with respect to each other.

## Discussion

### Cost-effectiveness analysis

Three metrics are relevant in this analysis for decision-making and policy concerning diagnostic testing for PCP: (a) proportion of PCP patients successfully treated, (b) proportion of well persons unnecessarily treated, and (c) the total diagnostic and treatment cost per life-year gained. An ideal test will maximize the first metric and minimize the second, at the smallest – and most feasible, for the implementing clinic or geographic region under consideration – value of the third. Because all laboratory-based diagnostic procedures considered in this analysis were highly specific, the effect of (b) is negligible for this analysis; thus, we presented the results as a function of (a) and (c).

Our results indicate that PCR methodologies are so sensitive that, specimen type notwithstanding, they represent the most cost-effective diagnostic options for PCP. When PCR methodologies are available, they mitigate the need for obtaining highly invasive specimens, such as BAL, which increase procedural sensitivity at substantial increases in cost. However, if both PCR and machinery for sputum induction are unavailable at a given site, the next-best option could be ES/TBO, which is relatively inexpensive and simple in terms of specimen collection and laboratory requirements for diagnosis. Although the use of chest x-ray alone for diagnosis can lead to the successful detection and treatment of high proportions of patients, the cost per life-year gained exceeds that of other equally-sensitive or more sensitive methods for diagnosing disease.

In general, the decision about which test is most useful in a given region will depend on the estimated prevalence of PCP among persons tested, local technical capacity, and available financial resources. Individual patient characteristics may affect decision-making, too; in an already-intubated patient, a BAL will be meaningfully cheaper than it would be among non-intubated patients, making the increased sensitivity in specimen collection more economical as well as practical (as an intubated patient or an infant will be unable to produce sputum). In addition, BAL might facilitate the detection of other respiratory pathogens besides *Pneumocystis*, such as TB or staphylococcus. Similarly, chest x-rays can provide information beyond the ability to evaluate a patient for signs consistent with PCP. However, for the diagnosis of PCP, providing a patient can produce sputum, the model presented suggests that there is little added value in carrying out a BAL over an induced sputum procedure.

There are several limitations to this analysis. First, data were not available on the sensitivity and specificity of all diagnostic procedures, creating a need to estimate some values. Even for procedures for which data were available, the degree of experience of an administering clinician or laboratory technician could affect the test's sensitivity or specificity. Second, indirect costs are not included in the model. The buildings, equipment, and technical know-how needed to carry out more advanced molecular diagnostics such as PCR are not currently in place in all countries. Where this capacity does exist, it may be unevenly distributed geographically and might not be accompanied by appropriate quality assurance measures. Start-up costs to implement these technologies could be prohibitive for some low-income countries, and in these areas comparisons of the cost-effectiveness of the various staining methods might be more useful than considerations about which PCR methodology is optimal. Finally, we did not account for differing diagnostic or treatment costs in different countries or among different patient groups, which could affect overall cost or cost-effectiveness of different diagnostic options. However, it is worth noting that, although the costs of all procedures might differ by country, the relative cost of procedures is unlikely to differ greatly.

### Other considerations

Existing international guidelines call for CTX prophylaxis of PCP in patients whose CD4+ T cell counts drop below 350 cells/mm [3]. CTX is also considered the treatment of choice for PCP. Because diagnostics are not available throughout much of the developing world and because CTX is relatively inexpensive and can be effective in the treatment of other respiratory pathogens in addition to PCP [88], [89], clinicians in developing countries frequently diagnose symptomatic patients empirically for PCP. While this method may capture a high proportion of patients with disease, it is highly nonspecific and thus may result in many patients without PCP being treated for the disease. Why should we consider this important? First, CTX has known toxic side effects: HIV-infected patients in particular are at risk of adverse reactions to CTX, including cutaneous reactions [90], fever, neutropenia, thrombocytopenia, transaminase elevation [91], meningitis [92] and anaphylaxis [93]. Second, non-judicious use of antimicrobials has long been recognized as a precursor to increased drug-resistance for a broad spectrum of pathogens. While reports of *Pneumocystis* resistance to CTX are infrequent, some do exist, and concern about resistance is increasing [94], [95], [96]. Perhaps as importantly, treatment for other pathogens, including *S. pneumoniae*, malaria, *Salmonella spp*, *Staphylococcus aureus*, and *Escherichia coli*, involve the use of CTX, and for these pathogens reports of CTX drug-resistance are common [97], [98], [99], [100], [101], [102], [103].

The cost-effectiveness of diagnostic testing improves in areas of higher disease prevalence; testing might become prohibitively expensive in areas with very low prevalence of disease. However, a diagnostic protocol that might seem financially unfeasible for certain regions might be more feasible than suspected if the prevalence of disease can be increased among the patients selected for testing. One way to optimize test utility is to use a clinical algorithm that improves the pre-test probability without incurring substantial numbers of false negatives. Although no such algorithm has been formally defined for PCP, clinical differences do exist between HIV-infected patients with PCP compared with other pneumonias; PCP patients have a more subacute onset of disease, ground glass infiltrates on CXR [104], lower oxygen saturation, lower CD4 cell counts, greater weight loss, more cyanosis, more severe dyspnea, and higher respiratory rates than non-PCP patients [15], [105]. Utilizing one or a combination of these metrics might be useful for increasing the prevalence of PCP among the population to be tested (e.g., increasing the pre-test probability), provided it did not miss substantial numbers of patients with PCP.

For any disease, when the cost of diagnosis exceeds the cost of treatment (such as with PCP), the cost-effectiveness of empiric diagnosis and treatment is directly proportional to the gap between the diagnostic and treatment costs; thus, when treatment costs are very low, it's nearly always more cost-effective to diagnose and treat patients empirically. In addition, because international guidelines call for at-risk patients to be on ART and CTX prophylaxis, the occurrence of PCP in a patient likely represents a failure of the local health system to provide sufficient opportunities for HIV patient care and treatment, an inability by the treating clinic to meet these standards, an inability by the patient to adhere to the recommended treatment regimen, or drug failure. Thus, one could argue that efforts should be focused on improving access to care for HIV patients or adherence to the standards laid out in international guidelines with respect to ART and CTX treatment, rather than on diagnosing the precise etiology of infections that could otherwise have been prevented. This is a valid argument and such efforts should be supported. However, given the suboptimal conditions that currently exist with respect to meeting these guidelines, there are benefits to accurate diagnosis, including improvements in the understanding of the true prevalence of disease, which is worthwhile for the purposes of prevention, control, and allocation of resources. This analysis is not intended to discourage PCP prophylaxis or diagnosis and treatment among symptomatic patients in the absence of a laboratory-based diagnosis, but rather to provide a basis for decisions on diagnostics for PCP, should an institution desire to implement diagnostic procedures. For these institutions, particularly in situations of high disease prevalence, we demonstrate that the elevated sensitivity and specificity of diagnosis enabled by the use of PCR technologies could justify the additional costs of obtaining and using them. A rough calculation demonstrates the power of replacing microscope-based technologies with PCR technologies for the diagnosis of PCP: in South Africa, the adult HIV infection rate is reported at 20%, with an estimated half a million new infections [106] and approximately 250,000 persons starting ART each year [107]. Assuming a PCP prevalence of ∼20% among HIV-infected persons starting ART (PCP prevalence among HIV-infected children is reported to be as high as 52% in South Africa [7], [8], [18]), approximately 50,000 persons would start ART with PCP. Using PCR technologies with expectorated sputum could result in the successful treatment of between 8,350 and 27,650 more PCP infections (i.e., result in 8,350–27,650 more life-years gained) than diagnosis with non-molecular-based technologies (or empiric diagnosis and treatment).

Diagnostic procedural decisions cannot, in practice, be simplified to numbers alone. Assuming clinicians were aware of the diagnostic qualities of each test, they could make decisions outside of the framework presented here, such as conducting sequential tests (for example, a highly sensitive test followed by a highly specific test) for diagnostic purposes. In addition, we realize that most clinicians do not have an array of diagnostic options at hand, and if a diagnostic protocol is to be implemented, it will be done at a clinic, hospital, or regional level. However, examples of molecular diagnostic technologies in resource-limited settings are increasingly reported, for example with tuberculosis diagnosis [108], [109], [110], [111]. The fixed costs associated with building structures and capacity to carry out these technologies will decrease in proportion to their utility as these technologies become cheaper and address to increasing arrays of pathogens. As more patients develop PCP and concerns about CTX drug resistance grow, it is worth considering whether changes in existing diagnostic paradigms are warranted for PCP. Choosing suboptimal diagnostic methodologies – or no laboratory-based methodologies at all, for empirically-diagnosed disease - for a treatable infection may no longer be justifiable, particularly in high-prevalence areas. In recognition of the enormous increases in diagnostic sensitivity available with more technologically complex procedures such as PCR, we would encourage policymakers, particularly those in regions where disease prevalence is high among the population tested, to consider prioritizing the development of the skills and infrastructure necessary to support improved diagnostic methods. It is our hope that this analysis can serve as a guide to help clinicians or policymakers make decisions about the best use of limited resources.

## Supporting Information

Table S1 Model inputs: Estimated personnel and time requirements and associated costs for specimen collection options for *Pneumocystis* pneumonia. *Estimates not available from laboratories; costs estimated by authors. BAL cost does not include cost of intubation. See Appendix S1 for working year and salary assumptions. †Time calculations based on procedure being performed on one patient at a time. ^1^Oral wash involves patients rinsing their oral cavities with a small volume of sterile saline and gargling for one minute before expectorating into a cup. ^2^Expectorated sputum involves asking a patient to inhale deeply several times before producing a deep cough from the chest. ^3^Induced sputum involves inhaling 3% sterile saline for 15–30 min using an ultrasonic nebulizer before asking the patient to expectorate sputum. ^4^Bronchoalveolar lavage involves instilling fluid into the lung and recovering the fluid using a bronchoscope.
(DOC)

Table S2 Model inputs: Personnel and time requirements and associated costs for laboratory procedures for diagnosis of *Pneumocystis* pneumonia. ¥Time estimated from starting sample to result ready to be reported. †Cost for personnel time is estimated as the amount of time a test takes excluding machine running times. We assumed that an average of five samples could be processed concurrently, dividing personnel-time costs by five. *Estimates not available from laboratories; values estimated by authors. CXR: Chest x-ray; DQ: Diff-Quick; GMS: Grocott's Methenamine Silver Stain; TBO: Toluidine Blue O; CW: Calcofluor white stain; IFA: Immunofluorescence microscopy assay; PCR: Polymerase chain reaction; nPCR: nested PCR; rtPCR: real-time (quantitative) PCR.
(DOC)

Table S3 Model inputs: cost of treatment, treatment failure rate, and prevalence of disease in the population.
(DOC)

Table S4 Sensitivity analysis: cost per life-year gained with variations in diagnostic procedure cost, sensitivity, specificity, treatment failure rates, and treatment costs. *Neither sensitivity nor specificity was increased beyond a value of 1.00. Procedures which in the base model were 0.90 or greater were capped at 1.00. CXR: Chest x-ray; DQ: Diff-Quick; GMS: Grocott's Methenamine Silver Stain; TBO: Toluidine Blue O; CW: Calcofluor white stain; IFA: Immunofluorescence microscopy assay; PCR: Polymerase chain reaction; nPCR: nested PCR; rtPCR: real-time (quantitative) PCR; Expect. sputum, expectorated sputum.
(DOC)

Appendix S1 Estimated salaries for laboratory workers, health care workers, and clinicians involved in patient care and diagnosis of PCP.
(DOC)
