# Supplementary material for: Cost-Effectiveness Analysis of Diagnostic Options for Pneumocystis Pneumonia (PCP)
Source: PLoS One. 2011 Aug 15;6(8):e23158. doi: 10.1371/journal.pone.0023158 (PMC3156114; doi:10.1371/journal.pone.0023158)
Supplement: Table S1 — Model inputs: Estimated personnel and time requirements and associated costs for specimen collection options for Pneumocystis pneumonia. *Estimates not available from laboratories; costs estimated by authors. BAL cost does not include cost of intubation. See Appendix S1 for working year and salary assumptions. †Time calculations based on procedure being performed on one patient at a time. 1Oral wash involves patients rinsing their oral cavities with a small volume of sterile saline and gargling for one minute before expectorating into a cup. 2Expectorated sputum involves asking a patient to inhale deeply several times before producing a deep cough from the chest. 3Induced sputum involves inhaling 3% sterile saline for 15–30 min using an ultrasonic nebulizer before asking the patient to expectorate sputum. 4Bronchoalveolar lavage involves instilling fluid into the lung and recovering the fluid using a bronchoscope. (DOC) [file pone.0023158.s001.doc]

Table S1. Model inputs: Estimated personnel and time requirements and associated costs for specimen collection options for *Pneumocystis* pneumonia.

| Specimen type | Personnel required | Time required | Cost for personnel time † | Cost of procedure / materials per specimen |
| --- | --- | --- | --- | --- |
| Oral wash^1^ | 1 nurse | 10 minutes | $1.96 | $0.20* |
| Expectorated^2^ sputum | 1 nurse | 15 minutes | $2.93 | $0.10* |
| Induced sputum^3^ | 1 physiotherapist | 30 minutes | $7.14 | $6.60 |
| BAL^4^ | 1 doctor | 60 minutes | $31.71 | $75.00* |
