# Supplementary material for: Cost-Effectiveness Analysis of Diagnostic Options for Pneumocystis Pneumonia (PCP)
Source: PLoS One. 2011 Aug 15;6(8):e23158. doi: 10.1371/journal.pone.0023158 (PMC3156114; doi:10.1371/journal.pone.0023158)
Supplement: Table S2 — Model inputs: Personnel and time requirements and associated costs for laboratory procedures for diagnosis of Pneumocystis pneumonia. ¥Time estimated from starting sample to result ready to be reported. †Cost for personnel time is estimated as the amount of time a test takes excluding machine running times. We assumed that an average of five samples could be processed concurrently, dividing personnel-time costs by five. *Estimates not available from laboratories; values estimated by authors. CXR: Chest x-ray; DQ: Diff-Quick; GMS: Grocott's Methenamine Silver Stain; TBO: Toluidine Blue O; CW: Calcofluor white stain; IFA: Immunofluorescence microscopy assay; PCR: Polymerase chain reaction; nPCR: nested PCR; rtPCR: real-time (quantitative) PCR. (DOC) [file pone.0023158.s002.doc]

Table S2. Model inputs: Personnel and time requirements and associated costs for laboratory procedures for diagnosis of *Pneumocystis* pneumonia.

| Diagnostic | Personnel required | Personnel time required¥ | Cost for personnel time† | Procedure / material costs, per specimen |
| --- | --- | --- | --- | --- |
| DQ | 1 technician | 45 minutes | $1.90 | $0.22 |
| GMS | 1 technician | 1.5 hours* | $3.81 | $0.20 |
| TBO | 1 technician | 15 minutes* | $0.63 | $0.10 |
| CW | 1 technician | 1 hour | $2.54 | $0.20 |
| IFA | 1 technician | 2.5 hours | $6.34 | $14.25 |
| PCR | 1 technician | 1 hour | $2.54 | $6.04 |
| nPCR | 1 technician | 1.5 hours | $3.81 | $6.31 |
| rtPCR | 1 technician | 2 hours | $5.08 | $8.56 |
