# Supplementary material for: Cost-Effectiveness Analysis of Diagnostic Options for Pneumocystis Pneumonia (PCP)
Source: PLoS One. 2011 Aug 15;6(8):e23158. doi: 10.1371/journal.pone.0023158 (PMC3156114; doi:10.1371/journal.pone.0023158)
Supplement: Table S3 — Model inputs: cost of treatment, treatment failure rate, and prevalence of disease in the population. (DOC) [file pone.0023158.s003.doc]

Table S3. Model inputs: cost of treatment, treatment failure rate, and prevalence of disease in the population.

| **Assumption** | **Value** |
| --- | --- |
| Cost of single 21-day course of oral cotrimoxazole | $2.77 |
| Treatment failure rate (including breakthrough and noncompliance which leads to drug failure) | 10% of treated patients |
| Prevalence of disease among patients tested (model independent variable) | 5% |
|  | 20% |
|  | 50% |
