# Supplementary material for: Cost-Effectiveness Analysis of Diagnostic Options for Pneumocystis Pneumonia (PCP)
Source: PLoS One. 2011 Aug 15;6(8):e23158. doi: 10.1371/journal.pone.0023158 (PMC3156114; doi:10.1371/journal.pone.0023158)
Supplement: Table S4 — Sensitivity analysis: cost per life-year gained with variations in diagnostic procedure cost, sensitivity, specificity, treatment failure rates, and treatment costs. *Neither sensitivity nor specificity was increased beyond a value of 1.00. Procedures which in the base model were 0.90 or greater were capped at 1.00. CXR: Chest x-ray; DQ: Diff-Quick; GMS: Grocott's Methenamine Silver Stain; TBO: Toluidine Blue O; CW: Calcofluor white stain; IFA: Immunofluorescence microscopy assay; PCR: Polymerase chain reaction; nPCR: nested PCR; rtPCR: real-time (quantitative) PCR; Expect. sputum, expectorated sputum. (DOC) [file pone.0023158.s004.doc]

Table S4. Sensitivity analysis: cost per life-year gained with variations in diagnostic procedure cost, sensitivity, specificity, treatment failure rates, and treatment costs.

|  |  |  | **Diagnostic procedure cost variation** | | **Diagnostic procedure sensitivity variation*** | | **Diagnostic procedure specificity variation*** | | **Treatment failure variation** | | **Treatment cost variation** | |
| --- | --- | --- | --- | --- | --- | --- | --- | --- | --- | --- | --- | --- |
| Diagnostic | Specimen collection | **Base model** | **Reduced by 50%** | **Increased by 100%** | **Reduced by 10%** | **Increased by 10%** | **Reduced by 10%** | **Increased by 10%** | **No failure** | **25% failure** | **Reduced by 90%** | **Increased by 100%** |
| CXR | None | $109 | $57 | $212 | $121 | $99 | $109 | $108 | $98 | $130 | $104 | $114 |
| Diff-Quick | Oral wash | $20 | $12 | $37 | $22 | $19 | $21 | $20 | $18 | $24 | $17 | $23 |
|  | Expect. sputum | $11 | $7 | $20 | $12 | $11 | $12 | $11 | $10 | $14 | $9 | $14 |
|  | Induced sputum | $29 | $16 | $55 | $32 | $27 | $29 | $29 | $26 | $35 | $26 | $32 |
|  | BAL | $232 | $117 | $460 | $257 | $211 | $232 | $232 | $208 | $278 | $229 | $235 |
| GMS | Oral wash | $34 | $19 | $65 | $38 | $31 | $35 | $34 | $31 | $41 | $31 | $37 |
|  | Expect.sputum | $21 | $12 | $39 | $23 | $19 | $22 | $21 | $19 | $25 | $18 | $24 |
|  | Induced sputum | $37 | $20 | $71 | $41 | $34 | $37 | $37 | $33 | $44 | $34 | $40 |
|  | BAL | $217 | $110 | $431 | $241 | $198 | $218 | $217 | $196 | $261 | $214 | $220 |
| TBO | Oral wash | $10 | $7 | $17 | $11 | $9 | $11 | $10 | $9 | $12 | $7 | $13 |
|  | Expect. sputum | $6 | $4 | $8 | $6 | $5 | $6 | $6 | $5 | $7 | $3 | $9 |
|  | Induced sputum | $25 | $14 | $47 | $27 | $23 | $25 | $25 | $22 | $30 | $22 | $28 |
|  | BAL | $213 | $108 | $424 | $237 | $194 | $214 | $213 | $192 | $256 | $211 | $217 |
| CW | Oral wash | $24 | $14 | $45 | $26 | $22 | $25 | $24 | $22 | $29 | $21 | $27 |
|  | Expect. sputum | $22 | $12 | $40 | $24 | $20 | $22 | $22 | $19 | $26 | $19 | $25 |
|  | Induced sputum | $39 | $21 | $75 | $43 | $36 | $40 | $39 | $35 | $47 | $36 | $42 |
|  | BAL | $224 | $114 | $445 | $249 | $204 | $225 | $224 | $202 | $269 | $222 | $227 |
| IFA | Oral wash | $157 | $80 | $311 | $174 | $143 | $158 | $157 | $141 | $188 | $154 | $160 |
|  | Expect. sputum | $95 | $49 | $187 | $105 | $87 | $96 | $95 | $86 | $114 | $92 | $98 |
|  | Induced sputum | $78 | $40 | $152 | $86 | $71 | $78 | $78 | $70 | $93 | $75 | $81 |
|  | BAL | $216 | $109 | $428 | $239 | $216 | $216 | $216 | $194 | $259 | $213 | $219 |
| PCR | Oral wash | $31 | $17 | $58 | $34 | $28 | $31 | $31 | $28 | $37 | $28 | $34 |
|  | Expect. sputum | $26 | $14 | $48 | $28 | $24 | $26 | $26 | $23 | $31 | $23 | $29 |
|  | Induced sputum | $39 | $21 | $75 | $43 | $36 | $39 | $39 | $35 | $47 | $36 | $42 |
|  | BAL | $189 | $96 | $375 | $210 | $189 | $189 | $189 | $170 | $227 | $186 | $192 |
| nPCR | Oral wash | $31 | $17 | $58 | $34 | $28 | $31 | $31 | $28 | $37 | $28 | $34 |
|  | Expect.sputum | $28 | $16 | $53 | $31 | $26 | $28 | $28 | $25 | $34 | $25 | $31 |
|  | Induced sputum | $40 | $22 | $77 | $44 | $40 | $41 | $40 | $36 | $48 | $37 | $43 |
|  | BAL | $193 | $98 | $382 | $214 | $193 | $193 | $192 | $173 | $231 | $190 | $196 |
| rtPCR | Oral wash | $38 | $21 | $72 | $42 | $35 | $38 | $38 | $34 | $45 | $35 | $41 |
|  | Expect. sputum | $36 | $20 | $70 | $40 | $34 | $37 | $36 | $33 | $44 | $34 | $40 |
|  | Induced sputum | $51 | $27 | $98 | $56 | $48 | $51 | $50 | $46 | $61 | $48 | $54 |
|  | BAL | $203 | $103 | $402 | $225 | $201 | $203 | $202 | $182 | $243 | $199 | $206 |
