# Supplementary material for: Cost-Effectiveness Analysis of Diagnostic Options for Pneumocystis Pneumonia (PCP)
Source: PLoS One. 2011 Aug 15;6(8):e23158. doi: 10.1371/journal.pone.0023158 (PMC3156114; doi:10.1371/journal.pone.0023158)
Supplement: Appendix S1 — Estimated salaries for laboratory workers, health care workers, and clinicians involved in patient care and diagnosis of PCP. (DOC) [file pone.0023158.s005.doc]

**Appendix S1.** Estimated salaries for laboratory workers, health care workers, and clinicians involved in patient care and diagnosis of PCP. Costs were converted from ZAR to USD based on 7.36 ZAR/1 USD and are based on a 260-day workyear, 8 hour workday.

**Salaries per annum***

Nurse: 180,000 ZAR ($24,456 USD)

Laboratory technician: 200,000 ZAR/yr ($27,174 USD)

Physiotherapist: 225,000 ZAR/yr ($30,571 USD)

Physician: 500,000 ZAR ($67,935 USD)

*Estimated salaries provided by Graeme Meintjes and Desiree DuPlessis of the National Institutes of Communicable Diseases, South Africa.
